# A systematic review of non-pharmacological interventions to improve nighttime sleep among residents of long-term care settings

**DOI:** 10.1186/s12877-018-0794-3

**Published:** 2018-06-18

**Authors:** Elizabeth Capezuti, Rana Sagha Zadeh, Kevin Pain, Aleksa Basara, Nancy Ziyan Jiang, Ana C. Krieger

**Affiliations:** 1 Hunter College School of Nursing and the Graduate Center, City University of New York, 425 E. 25th Street, New York, NY 10011 USA; 2 Design and Environmental Analysis, Cornell University, 170 Martha Van Rensselaer Hall, Ithaca, NY 14853-4401 USA; 3 Weill Cornell Medicine, Samuel J. Wood Library & C.V. Starr Biomedical Information Center, 1300 York Avenue Room, C-115, New York, NY 10065-4896 USA; 4 Department of Economics, Cornell University, 170 Martha Van Rensselaer Hall, Ithaca, NY 14853-4401 USA; 5 Health Design Innovations Lab, Department of Design & Environmental Analysis, Cornell University, 170 Martha Van Rensselaer Hall, Ithaca, NY 14853-4401 USA; 6 Departments of Medicine, Neurology and Genetic Medicine, Weill Cornell Medical College, Cornell University, 425 E. 61st St., 5th Floor, New York, NY 10065 USA

**Keywords:** Sleep, Circadian rhythms, Non-pharmacological intervention, Nursing homes

## Abstract

**Background:**

Disturbances in sleep and circadian rhythms are common among residents of long-term care facilities. In this systematic review, we aim to identify and evaluate the literature documenting the outcomes associated with non-pharmacological interventions to improve nighttime sleep among long-term care residents.

**Methods:**

The Preferred Reporting Items for Systematic Reviews guided searches of five databases (MEDLINE, Embase, CINAHL, Scopus, and Cochrane Library) for articles reporting results of experimental or quasi-experimental studies conducted in long-term care settings (nursing homes, assisted-living facilities, or group homes) in which nighttime sleep was subjectively or objectively measured as a primary outcome. We categorized each intervention by its intended use and how it was administered.

**Results:**

Of the 54 included studies evaluating the effects of 25 different non-pharmacological interventions, more than half employed a randomized controlled trial design (*n* = 30); the others used a pre-post design with (*n* = 11) or without (*n* = 13) a comparison group. The majority of randomized controlled trials were at low risk for most types of bias, and most other studies met the standard quality criteria. The interventions were categorized as environmental interventions (*n* = 14), complementary health practices (*n* = 12), social/physical stimulation (*n* = 11), clinical care practices (*n* = 3), or mind-body practices (*n* = 3). Although there was no clear pattern of positive findings, three interventions had the most promising results: increased daytime light exposure, nighttime use of melatonin, and acupressure.

**Conclusions:**

Non-pharmacological interventions have the potential to improve sleep for residents of long-term care facilities. Further research is needed to better standardize such interventions and provide clear implementation guidelines using cost-effective practices.

**Electronic supplementary material:**

The online version of this article (10.1186/s12877-018-0794-3) contains supplementary material, which is available to authorized users.

## Background

Many long-term care residents have sleep and circadian rhythm disturbances [1, 2] due to advanced age, the effects of certain chronic illnesses and medications, declining brain health, diminished mobility, and other causes [3, 4]. Therefore, the American Geriatrics Society and the National Institute on Aging now recognize a geriatric syndrome in which physical and mental risk factors overlap to increase risk for sleep and circadian disturbances. The relationship between some risk factors and sleep disturbance is often bidirectional [3]. Numerous negative consequences are associated with sleep disturbances, including increases in cognitive decline, metabolic disease, high blood pressure, cardiovascular disease mortality, frailty, impaired quality of life, and hypersensitivity to pain [3].

Long-term care residents have a high prevalence of multimorbidity that includes both chronic physical (e.g., advanced cardiovascular or pulmonary disease, arthritis) and mental (e.g., dementia, depression) illnesses that are associated with sleep and circadian rhythm disturbances [4, 5]. More than a quarter of nursing-home residents and approximately 70% of assisted-living facility residents have been diagnosed with dementia [5, 6], and almost half of those will have sleep disturbance [7, 8]. Sleep disturbance in dementia patients is associated with anxiety and behavioral symptoms of agitation, aggressiveness, and disinhibition [7]. Sleep disturbances and accompanying symptoms often lead providers to prescribe psychoactive medications, including hypnotics. Sedative-hypnotic pharmaceuticals are commonly used for assisted-living facility residents with dementia [9]. Similarly, about 47% of nursing-home residents with dementia are prescribed sedative-hypnotics, especially when displaying anxiety and agitation [10].

However, both benzodiazepines and non-benzodiazepine receptor agonist hypnotics have been associated with an increased risk of fall and fractures in older adults [11–13]. These challenges reinforce the need to consider non-pharmacological approaches in the unique physical environment and institutional milieu of long-term care facilities [14]. The potential for sleep or circadian rhythm disturbance is linked to an increase in vulnerability to environmental challenges in these facilities [1]. For some residents, a non-stimulating environment may lead to excessive daytime and early evening napping that would exacerbate sleep disturbances. Others may experience an over-stimulating nighttime environment due to light and noise or exposure to disruptive behaviors, including pain, discomfort, repetitive vocalizations, and wandering. Staff routines such as nighttime incontinence care are also disruptive to sleep maintenance [15].

The problems associated with existing pharmacological treatments, coupled with institutional environments that can further disrupt sleep, mean that non-pharmacological interventions should be considered to help prevent or manage sleep disturbance [16]. Consistent with recommendations of the American Geriatrics Society and the National Institute on Aging [3], the objective of this systematic review is to identify and evaluate the literature documenting the outcomes associated with non-pharmacological interventions to improve nighttime sleep among long-term care residents.

## Methods

Our original intent for this systematic review was to evaluate the literature addressing non-pharmacological interventions to promote sleep among adults across all institutional settings. As described below, our search was adjusted to focus on long-term care settings due to important differences between acute-care and residential settings, such as high medical acuity and short length of stay.

### Search methodology

The Preferred Reporting Items for Systematic Reviews and Meta-Analyses Statement was used to guide this review [17]. A library specialist (KP) performed systematic searches of MEDLINE (Ovid), Embase (Ovid), CINAHL (EBSCOhost), Scopus (Elsevier), and the Cochrane Library (Wiley) between August and October of 2016, with weekly search updates for all five databases through December 2016. Major search terms for all databases were represented by both controlled vocabulary and keywords (Table 1) on the topic of sleep quality in institutional healthcare settings. Where appropriate, searches were restricted to human, adult, English-language studies but were not otherwise limited by study design or date of publication. Specific inclusion and exclusion criteria are listed in Table 2 for the original systematic review. Studies were then further limited to those located in nursing homes, assisted living facilities, and other long-term care facilities. The complete search strategies are available in the supplemental materials (Additional file 1: Table S1- Search Strategies-BMC).

**Table 1:** Search terms

	Field	Key words
	Title	(sleep$ adj2 (disrupt$ or disturb$ or impair$ or interrupt$ or depriv$ or lack or poor or problem$))
OR	Title	(sleep$ adj2 (quality or quantity or duration or time$ or timing or pattern$ or rhythm$ or promotion or hygiene or efficiency or cycle$ or onset or health$ or hour$ or phase$ or support or help or initiat$))
OR	Title	insomnia$
OR	Title	circadian
OR	Title	(sleep$ adj5 “biological clock$”)
AND	All Text	(hospital$ or inpatient$ or institutional$ or “intensive care” or ward$ or hospice$ or “nursing home$” or “assisted living” or palliative or “end of life” or “end-of-life” or terminal or “health facilit$” or “residential facilit$” or icu or “critical care”)

**Table 2:** Inclusion and exclusion criteria

Inclusion	Exclusion
English-language articles	Articles with only partial content in English
Intervention studies published in peer-reviewed journals	Dissertations, presentation/poster abstracts, brief reports in letters to the editors, editorials/commentaries, literature reviews, or meta-analyses
Non-pharmacological interventions, including food supplements and melatonin, conducted within or outside the United States	Pharmacological interventions, models of care (e.g., palliative care team), or respiratory interventions
Experimental or quasi-experimental design	Single case studies or cross-sectional design
Adult participants, including those with dementia	Participants being treated for a medical sleep disorder or those with a psychiatric disorder such as depression, schizophrenia, or addiction disorder
Any setting, including hospitals, long-term care settings (nursing homes, assisted-living facilities, or group homes), in-patient hospice or other institutional settings, or simulated hospital environments	Community settings including those involving interventions from home caregivers, outpatient clinics, dialysis centers, or adult foster homes, as well as psychiatric in-patient facilities
Nighttime sleep as a subjective (self-reported) or objective outcome (primary or secondary)	Sleep not measured with tool

### Study selection, data extraction, and analysis

After the removal of duplicates, the final set of 6747 articles was transferred to Covidence software for synthesis of the literature data. Two review authors (AB and NJ) independently assessed the eligibility of the retrieved articles by title and abstract. A third author (EC, RZ or AK) resolved all conflicts, with a total of 6302 articles excluded. The library specialist downloaded full-text versions of the remaining 445 articles. Teams of two review authors (AB, NJ, RZ or EC) then independently assessed the eligibility of the full-text articles. Any conflicts at this stage were resolved by another author (AK). Applying the exclusion criteria to this body of literature, another 311 articles were removed. The major reasons for exclusion, in order of frequency, were literature review, presentation abstract, wrong patient population, editorial, wrong outcomes (not sleep), and wrong setting.

**Fig. 1:**
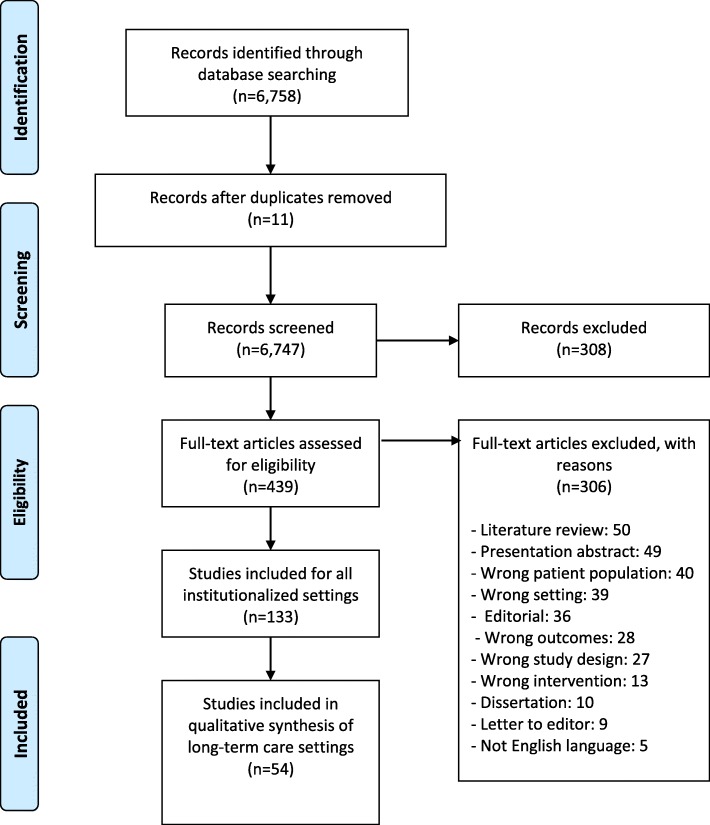
PRISMA flow diagram

The resulting set of 134 articles was split between hospital-based studies (*n* = 79) and the final set of 54 articles focusing on long-term care settings that we used in this analysis. We used the details from the selection process in Covidence to complete a PRISMA flow diagram (Fig. 1) [17] and exported all titles and abstracts to EndNote software. Two review authors (AB and NJ) independently extracted study characteristics in Microsoft Excel and then summarized the characteristics of the included studies. A second review author (RZ or EC) checked the accuracy of the table against the original articles.

We revised the intervention coding we used for an integrative review conducted by this research team that focused on non-pharmacological intervention for sleep in patients with advanced serious illness for this reviews’ population of long term care residents [18]. We used an iterative approach to code interventions. First, we examined the intent of the intervention, such as what risk factor for sleep disturbance was targeted for reduction or elimination (e.g., daytime physical exercise to address excessive daytime napping). The categories that emerged from the data were mostly interventions manipulated by staff or study personnel: environmental factors (external to the resident), complementary health practices (touch and oral supplements), social and physical stimulation (activities for exercise or engaging the resident cognitively), and clinical care practices (reducing sleep disruptions). The final category, mind-body practices, are those in which residents actively participate and include activities such as self-relaxation and meditation.

After evaluation of the interventions and intended outcomes, we then determined their overall effect on actual outcomes related to sleep disturbance. We categorized interventions as having a positive effect, a mixed effect (some positive and some inconclusive outcomes), no effect, or a negative effect. Our overall summary of the findings was based on nighttime sleep outcomes, although some studies also reported daytime sleep results. The risk of bias in the included studies was evaluated using the Cochrane Risk of Bias methodology for randomized clinical trials [19] and the Summary Quantitative Studies and Critical Appraisal Checklist for all other studies [20].

## Results

Study characteristics are summarized in Tables 3 and 4 (multicomponent). Of the 54 studies, more than half employed a randomized controlled trial (RCT) design (*n* = 30); the others used a pre-post design with (*n* = 11) or without (*n* = 13) a comparison group.

**Table 3:** Characteristics of Included Studies (except multi-component interventions; *n* = 43)

First author, Year	Design, Number of groups, and Study type	Setting (number of facilities), Number of participants, Mean age, % male, and Inclusion/exclusion criteria	Description of intervention	Effect (positive, mixed, none, or negative), Measurement of sleep, Main finding(s)
Clinical care practices (*n* = 3)				
Kim, 2016 [73]	Quasi-experimental pre-post intervention with 3 groups (intervention, comparison with placebo of 36.5 ° C water and control)	Nursing home (*n* = 1), N = 30, mean age 85.9, 20% men	Adjust core body temperature: 30 min of warm (40 ° C) foot baths in the evening daily for 4 weeks	None; Actigraphy; No significant differences in total sleep amount, efficiency, or latency among the 3 groups
O’Rourke, 2001 [25]	Quasi-experimental pre-post intervention without comparison	Assisted-living facility (*n* = 2), *N* = 18, mean age 84.5, 22% men, with incontinence	Minimize clinical disruptions: 15 consecutive days alternating every 5 days between usual nighttime rounds and non-disruptive nighttime care	Positive; 24-h monitoring at 30-min intervals; Significant improvement in total nighttime sleep by 30 min (*p* = 0.01)
Matthews, 1996 [41]	Quasi-experimental pre-post intervention without comparison group	Nursing home (*n* = 1), *N* = 33, mean age 84.2, 36.3% men, with dementia	Individualize care: Four 4-week phases of changes in staff behavior from task-oriented to individualized (client-centered) care	None; Sleep subscale of Dementia Mood Assessment Scale; Nighttime sleep did not change significantly
Mind-body practices (*n* = 3)				
Chen, 2010 [22]	Quasi-experimental pre-post intervention with comparison	Assisted-living facility (*n* = 2), *N* = 55, mean age 75.4, 47.3% men	Relaxation techniques: 70-min sessions of Silver Hatha Yoga (adapted for older population) 3 times per week for 6 months	Positive; Pittsburgh Sleep Quality Index; Overall sleep quality significantly improved, and sleep disturbances and daytime dysfunction decreased significantly (*p* = 0.05)
Örsal, 2014 [58]	Quasi-experimental pre-post intervention with comparison	Nursing home (*n* = 1), *N* = 64, mean age 75.8, 57.8% men	Relaxation techniques: Progressive muscle relaxation exercises each night between 9 pm and 12 MN (for a total of 30 min/week) each night × 7 days	Positive; Pittsburgh Sleep Quality Index; Quality of sleep improved significantly (*p* = 0.000)
El Kady, 2012 [50]	Quasi-experimental pre-post intervention without comparison	Nursing home (*n* = 4), *N* = 210, mean age 72.2, 46.2% men, with sleep problems	Cognitive-behavioral therapy: Four 30-min sessions of cognitive behavioral sleep therapy using sleep hygiene education and stimulus control techniques	Positive; Pittsburgh Sleep Quality Index; Statistically higher improvement in sleep quality (percentage of poor sleepers decreased from 63.3 to 46.2%)
Social and physical stimulation (*n* = 11)				
Kuck, 2014 [51]	RCT clustered by nursing home	Nursing home (*n* = 20), *N* = 85, mean age 83.9, 75.5% men, with sleep problems	Combination (social/physical): 2 days per week of both two 45-min sessions of social activity and two sessions of physical exercise (balance & muscle strengthening) for 8 weeks	Mixed Actigraphy & Insomnia Severity Index; No improvement by actigraphy measures in the intervention group but subjective sleep quality increased post intervention (*p* = 0.04)
Lorenz, 2012 [55]	RCT, 4 groups (3 intervention and 1 control)	Nursing home (*n* = 13), *N* = 193, mean age 81.4, 36% men	Combination (social/physical): 3 intervention groups of exercise (3 days physical resistance training and 2 days walking per week), individualized social activity (1 h per day 5 days per week), or both for 7 weeks	None; Polysomnography; No relationship between change in everyday function (from interventions) and change in sleep parameters
Richards, 2011 [26]	RCT, 4 groups (3 intervention and 1 control)	Nursing home (*n* = 10) and assisted-living centers (3), *N* = 165, mean age 81.8, 39.9% men	Combination (social/physical): 3 intervention groups of exercise (3 days physical resistance training and 2 days walking per week), individualized social activity (1 h per day 5 days per week), or both for 7 weeks	Positive; Polysomnography; Group receiving both treatments showed a significantly greater increase in total nocturnal sleep time and sleep efficiency over the control condition, but the exercise and social activity alone groups did not
Richards, 2001 [42]	Quasi-experimental pre-post intervention without comparison	Nursing home (*n* = 1), *N* = 5, mean age 76.2, 100% men, with dementia	Social and cognitive activity: 15–30 min of individualized activity for 1 to 2 h per day for 3 days	Positive; Actigraphy; Percent of nocturnal time asleep significantly increased (*p* < 0.01)
Richards, 2005 [43]	RCT	Nursing home (*n* = 7), *N* = 139, mean age 79, 51.8% men, with dementia	Social and cognitive activity: 1–2 h of individualized social activity for 21 days	Mixed; Actigraphy; Significantly reduced minutes to sleep onset, significantly reduced minutes awake, and increased sleep among those with baseline poor sleep but not for total group; sleep efficiency not improved
Thodberg, 2015 [46]	Quasi-experimental pre-post intervention (dog visit) with comparison (robot seal or toy cat)	Nursing home (*n* = 4), *N* = 100, mean age 85.5, with dementia	Social and cognitive activity: 10-min biweekly visits by an “animal” (dog, robot seal, or toy cat) for 6 weeks	Mixed; Actigraphy; Sleep duration increased in the third week for the dog group compared to the robot seal or toy cat (*p* = 0.01); no effects were found in the sixth week or after the visit period had ended
Alessi, 1995 [33]	Quasi-experimental pre-post intervention with comparison	Nursing home (*n* = 7), *N* = 65, mean age 84.8, 85% men, with urinary incontinence or physically restrained	Physical exercises: Exercises (transfers, walking, and rowing) performed every 2 h (8 am-4 pm) 5 days a week for 9 weeks	None; Actigraphy; No significant improvement in nighttime or daytime sleep
Chen, 2015 [57]	RCT (clustered by nursing home)	Nursing home (*n* = 10), *N* = 127, mean age 79.2, 50.9% men, wheelchair bound	Physical exercises: 40-min elastic-band exercises (in wheelchair) 3 times per week for 6 months	Positive; Pittsburgh Sleep Quality Index; Intervention group had significantly longer sleep duration at 3 and 6 months and overall better sleep quality at 6 months
Eggermont, 2010 [67]	RCT	Nursing home (*n* = 19), *N* = 79, mean age 84.3, 20.3% men, with dementia	Physical exercises: Five 30-min walking sessions per week for 6 weeks (total of 30 sessions)	None; Actigraphy; No significant improvement in nighttime restlessness, sleep efficiency, number of waking bouts, or daytime activity
Taboonpong, 2010 [32]	Quasi-experimental pre-post intervention with comparison	Elderly residential center (*n* = 2), *N* = 50, 58% men	Physical exercises: Tai Chi exercise at least 3 times a week for 22 min for 12 weeks	Positive; Pittsburgh Sleep Quality Index; Significant improvement in sleep quality (*p* < 0.01)
Lee, 2008 [23]	Quasi-experimental pre-post intervention without comparison	Assisted-living facility (*n* = 1), *N* = 23, with dementia	Physical activity: Indoor gardening twice daily for 4 weeks	Positive; 24-h sleep diary; Significant improvement in wake after sleep onset, nocturnal sleep time, and sleep efficiency
Complementary health practices (*n* = 12)				
Soden, 2004 [29]	RCT (3 groups: aromatherapy and massage, massage, or control)	Assisted-living facility (*n* = 3), *N* = 42, 24% men	Combination (touch and aromatherapy): Massage with lavender oil and/or massage with inert oil, each for 30 min for four weeks	Mixed; Verran and Snyder-Halpern Sleep Scale; Statistically significant improvement in the massage (*p* = 0.02) and combined massage (*p* = 0.03) groups but not for the aromatherapy and massage group only
Gehrman, 2009 [68]	RCT	Nursing home (*n* = 1), *N* = 41, mean age 82.9, 31.7% men; with dementia	Oral supplement: Melatonin (8.5 mg immediate release and 1.5 mg sustained release) administered at 10 pm for 10 consecutive nights	None; Actigraphy; No significant differences between the groups for nighttime or daytime sleep
Rondanelli, 2011 [28]	RCT	Assisted-living facility (*n* = 1), *N* = 43, mean age 78.3, with insomnia	Oral supplement: Melatonin (5 mg) plus dietary supplement (magnesium 225 mg and zinc 11.25 mg) every day for 8 weeks	Positive; Pittsburgh Sleep Quality Index; Significantly better sleep (*p* < 0.001)
Valtonen, 2005 [47]	Quasi-experimental pre-post intervention with comparison, crossover design (3 groups: 2 intervention and 1 control)	Nursing home (*n* = 2), *N* = 81, mean age 82.8, 21% men, mild cognitive impairment	Oral supplement: 8 weeks of melatonin-rich (5–20 mg/day) milk then 8 weeks of normal milk (and conversely for the other intervention group)	Mixed; Sleep questionnaire; In one intervention group, sleep quality, morning activity, and evening activity all increased significantly (*p* < 0.001) when milk was consumed in the evening
Braun, 1986 [62]	Quasi-experimental pre-post intervention without comparison	Nursing home (*n* = 1), *N* = 6, mean age 85, 0% men	Touch: 5 min of talking and 5 min of therapeutic touch 6 in. above the solar plexus	Positive; Visser’s Sleep Quality Assessment; Improved sleep quality
Chen, 1999 [21]	RCT	Nursing home (*n* = 1), *N* = 84, mean age 79, 61.9% men	Touch: 15 min of acupressure, consisting of 5 min of finger massage and 10 min of acupoint massage between 1 pm and 10 pm 5 days per week for 3 weeks	Positive; Pittsburgh Sleep Quality Index; Significantly more positive sleep including quality, latency, duration, efficiency; reduced disturbances of sleep; and frequencies of nocturnal awakening and night wakeful time.
Harris, 2012 [69]	RCT	Nursing home (*n* = 4), *N* = 40, mean age 86, 22.5% men, with dementia	Touch: 3-min slow-stroke back massage at bedtime for 2 nights	None; Actigraphy; No significant increase in minutes of nighttime sleep
Nelson, 2010 [74]	RCT	Nursing home (*n* = 4), *N* = 28, mean age 69.5, 57.1% men	Touch: 15-min massage to head, neck shoulders, and back between 8 pm and 10 pm every night and 7 days	None; Observed 3 participants asleep following the intervention
Reza, 2010 [59]	RCT (3 groups: intervention, sham, and control)	Nursing home (*n* = 1), *N* = 77, mean age 75.2, 53.2% men	Touch: 3 sessions of acupressure (hands, head, ears, and feet) per week for 4 weeks	Positive; Pittsburgh Sleep Quality Index; Compared to controls, the acupressure group had significantly positive subjective sleep quality, sleep latency, sleep duration, habitual sleep efficiency, sleep sufficiency, and reduced sleep disturbance. No differences between the sham and control groups.
Simoncini, 2015 [60]	Quasi-experimental pre-post intervention without comparison	Nursing home (*n* = 2), *N* = 129, mean age 82.7	Touch: Daily acupressure of 8 h continuously on the HT7 acupoint with a patch device for 8 weeks	Positive. Pittsburgh Sleep Quality Index; Significant improvement in ability to fall asleep and quality of sleep
Sun, 2010 [30]	RCT	Assisted-living facility (*n* = 2), *N* = 50, mean age 70.48, 64% men, with insomnia	Touch: 5 s of acupressure on HT7 acupoint of both wrists followed by 1-s rest repeated for 5-min before bedtime for 5 weeks	Positive; Athens Insomnia Scale-Taiwan Form; Significant improvement in sleep at 6 weeks post-intervention (*p* = 0.002)
Van Someren, 1998 [48]	RCT	Nursing home (*n* = 1), *N* = 14, mean age 84, 7.1% men, with early dementia	Touch: TENS (transcutaneous electrical nerve stimulation) between the shoulder blades for 30 min per day between 4 pm and 6 pm, 5 days a week for 6 weeks	Positive; Actigraphy; Post-treatment mean was significantly higher in the treatment group than both the pretreatment mean (*p* = 0.03) and the follow up mean (*p* = 0.03)
Environmental intervention (*n* = 14)				
Ancuelle, 2015 [56]	Quasi-experimental pre-post intervention without comparison	Nursing home (*n* = 1), *N* = 38, mean age 78.4, 44.7% men, with musculoskeletal pain	Ergonomic adjustment: 4 weeks of medium-firm mattress use	None. Pittsburg Sleep Quality Index and subsample with actigraphy. Sleep not significantly improved (*p* = 0.245).
Akyar, 2013 [49]	Quasi-experimental pre-post intervention without comparison	Nursing home (*n* = 1): *N* = 24, mean age 80, 33.3% men, with poor sleep quality	Increased light: 30 min of morning bright light (10,000 lx) for 30 days	Positive; Pittsburg Sleep Quality Index; Immediately and 4 weeks post-intervention, there was significant improvement on all sleep outcomes (*p* < 0.001)
Ancoli-Israel, 2002 [36]	RCT, 3 intervention groups (morning v. evening light) v. sleep restriction or comparison	Nursing homes (*n* = 2), *N* = 77, mean age 85.7, 24.7% men with dementia	Increased light: Bright light box (2500 lx) from 5:30 pm to 7:30 pm or 9:30 am to 11 am or daytime sleep restriction comparison with dim (50 lx) red light from 5:30 pm-7:30 pm for 10 days	None; Actigraphy; No improvements in nighttime sleep or daytime alertness in any of the treatment groups. (Note: Daytime restriction is comparison group.)
Ancoli-Israel, 2003 [35]	RCT, 3 groups (intervention of daytime or evening bright light with comparison of dim red light)	Nursing home (*n* = 2), *N* = 92, mean age 82.3, 31.5% men, with late-stage Alzheimer’s disease	Increased light: Either morning or evening bright light box (2500 lx) compared with morning dim (< 300 lx) red light for 10 days	Mixed; Actigraphy; Duration of maximum sleep bout significantly increased from 64.9 min to 88.4 min in the morning and evening bright light group. However, there was no effect on total sleep time or on night or day wake time.
Burns, 2009 [37]	RCT	Nursing home (*n* = 2), *N* = 48, mean age 83.5, 33% male; with dementia & agitation	Increased light: 2-h (10 am to 12 pm) bright light therapy (10,000 lx) for 2 weeks	None; Actigraphy; Mean duration of nocturnal sleep improved but not significantly
Calkins, 2007 [38]	RCT	Nursing home (*n* = 3), *N* = 17, 11.8% men, with dementia	Increased light: Outdoor daylight exposure for 30 min for 2 weeks	None; Actigraphy and Pittsburgh Sleep Quality Index; No significant improvement in sleep
Castor, 1991 [63]	Quasi-experimental pre-post intervention without comparison	Nursing home (*n* = 1), *N* = 12, mean age 70, 100% men	Increased light: Twice daily exposure (1 h in morning and 1 h in afternoon) to sunlight for 1 week	Positive; Nursing Assessment of Sleep; Significant improvement in uninterrupted sleep (*p* = 0.003) and mean sleep hours per 24 h (*p* = 0.052), as well as a decrease in night wake hours (*p* = 0.007)
Dowling, 2005 [40]	RCT	Assisted-living Facility (*n* = 2), *N* = 46, mean age 84, 22% men, with severe dementia	Increased light: 1 h of bright morning light (9:30 am to 10:30 am; 2500 lx) 5 days per week for 10 weeks	None; Actigraphy; No significant improvement in nighttime sleep efficiency, sleep time, wake time, or number of awakenings
Fetveit, 2003 [61]	Quasi-experimental pre-post intervention without comparison	Nursing home (*n* = 1), *N* = 11, mean age 86.1, 9.1% men, with dementia	Increased light: 2 h of morning (8 am to 11 am; 6000–8000 lx) bright light per day for 2 weeks	Positive; Actigraphy; Waking time within nighttime sleep reduced by 2 h and sleep efficiency improved from 73 to 86%
Fetveit, 2004 [64]	Quasi-experimental pre-post intervention without comparison	Nursing home (*n* = 1), *N* = 11, mean age 86.1, 9.1% men, with dementia	Increased light: 2 h of bright morning light (6000–8000 lx) per day for 2 weeks	Mixed; Actigraphy; Sleep efficiency remained higher than baseline for 4 weeks and sleep onset latency remained significantly reduced for 12 weeks
Figueiro, 2014 [53]	Quasi-experimental pre-post intervention without comparison	Nursing home (*n* = 1), *N* = 14, mean age 86.9, 37.7% men, with dementia	Increased light: 4 weeks of blush-white lighting (luminaires) in residents’ rooms with timer (from waking until 6 pm) for 4 weeks	Mixed; Daysimeter; Significant improvement in sleep efficiency (80 to 84%, *p =* 0.03) and sleep time (431 min to 460 min, *p* = 0.03) but not in sleep latency
Koyama, 1999 [71]	Quasi-experimental pre-post intervention without comparison	Nursing home (*n* = 2), *N* = 6, with dementia	Increased light: 1 or 2 h of late morning bright light (4000 lx)	None; sleep observation diary; Nighttime sleep maintained in 3 participants
Lyketsos, 1999 [24]	RCT	Assisted-living facility (*n* = 1), *N* = 8, mean age 80.8, 6.7% men, with dementia and agitated behaviors	Increased light: 1 h of morning bright light (10,000 lx) therapy for 4 weeks	Positive; sleep observation log, 8 pm-8 am; Statistically significant improvement in sleep duration from 6.4 h to 8.1 h (*p* < 0.05)
Wu, 2015 [31]	Quasi-experimental pre-post intervention with comparison (clustered by unit)	Assisted Living Facility (*n* = 1) *N* = 65, mean age 80, 57.1% men	Increased light: 30 min of morning (9:30 am–10 am) bright light (10,000 lx) therapy 3 times per week for 4 weeks	Mixed; sleep diary; Significant decrease in sleep disruptions in the experimental group from week 1 to week 4 (*p* = 0.02) but no significant difference between treatment and control groups

*RCT* Randomized controlled trial

**Table 4:** Characteristics of multi-component studies (*n* = 11)

					Environment		CHP	SPS	CCP		
First author, Year)	Design, Number of groups, and type	Setting (number of facilities), Number of participants, Mean age, % Male, Inclusion/exclusion criteria	Description of intervention	Effect (positive, mixed, none, or negative), Measurement of sleep, Main finding(s)	Light	Noise	Melatonin	Physical activity	Individual care	Less disruptions	Mange Sleep wake
Alessi, 1999 [34]	RCT	Nursing home (*n* = 1); *N* = 29, mean age 88.3, 10% male, with incontinence	Daytime physical activity and nighttime program to decrease noise and decrease sleep-disruptive nursing care practices 5 days per week for 14 weeks	Positive; Actigraphy; Nighttime sleep increased from 51.7 to 62.5% (*p* = 0.045)		X		X			X
Alessi, 2005 [65]	RCT	Nursing home (*n* = 4): *N* = 118, mean age 86.8, 45% men	Efforts to decrease daytime in-bed time, provide daily sunlight exposure for at least 30 min, increase physical activity, structure bedtime routines, and decrease nighttime noise and light for 5 consecutive days and nights	Mixed; Actigraphy; Modest decrease in nighttime awakenings (*p* = 0.04) but no effect on % of nighttime sleep or number of nighttime awakenings	X	X		X		X	X
Connel, 2007 [39]	RCT	Nursing home (*n* = 1), *N* = 20, mean age 79.7, 95% male, with dementia	1 h of group outdoor structured activity for 10 days (compared with indoor structured activity)	Positive; Actigraphy; Maximum sleep duration increased and total sleep minutes significantly increased	X			X			
Dowling, 2008 [66]	RCT	Nursing home (*n* = 2), *N* = 50, mean age 86, 14% men, with dementia	Melatonin 5 mg & 1 h of morning bright light (≥ 2500 lx) for 5 mid-week days per week for 10 weeks	None; Actigraphy; No significant impact on sleep	X		X				
Gammack, 2009 [72]	RCT	Nursing homes (*n* = 1), *N* = 24, mean age 79.5, 37.5% men, without dementia	60-min outdoor morning (between 7 am-12 pm) light and structured recreational activity for 21 days	None; Medical Outcome Study Sleep Scale; No differences in sleep scores between treatment and controls	X			X			
Ito, 2001 [70]	Quasi-experimental pre-post intervention with comparison (bright light only)	Nursing home, *N* = 28, mean age 78.3, 42.9% men, with Alzheimer’s disease	Daily Vitamin B12 (1.5 mg for 2 weeks then 3.0 mg for 2 weeks) and 2-h morning (9 am–11 am) bright light (3000 lx) therapy for 4 weeks	None; Actigraphy; No significant improvement in nighttime sleep outcomes	X		X				
Martin, 2007 [52]	RCT	Nursing home (*n* = 4) *N* = 118, mean age 87.05, 78% men, with sleep disruption	Exposure to outdoor bright light (at least 20,000 lx), efforts to keep residents out of bed during the day, bedtime routine, efforts to decrease nighttime noise and light, and structured physical activity for 10 min to 15 min 3 times per day for 5 days	None; Actigraphy; Significant change only in the active phase of the rest/activity rhythms. Not able to significantly reduce nighttime noise and light.	X			X			X
Ouslander, 2006 [54]	RCT, clustered by facility	Nursing home (*n* = 8), *N* = 160, mean age 83.2, 25% men	Daytime activities, keep residents out of bed, evening bright light, consistent bedtime routine, nighttime care routines to minimize disruption, and strategies to reduce nighttime noise for 17 days	None; Actigraphy and polysomnography; No improvement in nighttime sleep	X	X		X		X	X
Riemersma-Van Der Lek, 2008 [27]	RCT, clustered by facility with 4 groups (bright light, melatonin, combination, or none)	Assisted-living facilities (*n* = 12), *N* = 189, mean age 85.8, 10% men, with dementia	Whole-day bright light (10,000 lx) and 2.5 mg melatonin for a mean of 15 months	Positive; Actigraphy; Combined treatment (light and melatonin) significantly ameliorated nocturnal restlessness, reduced awakenings, and increased sleep efficiency. Melatonin shortened sleep onset latency and increased sleep duration.	X		X				
Schnelle, 1998 [44]	Quasi-experimental pre-post intervention with comparison	Nursing home (*n* = 4), *N* = 92, mean age 87.3, 19% men, with incontinence	Individualized nighttime incontinence care (every 2 or 4 h and when awake) and minimized sleep disruption for 5 nights	Positive; Actigraphy; Significant reduction in awakenings due to light and sound (*p* < 0.001)	X	X			X	X	
Schnelle, 1999 [45]	RCT	Nursing home (*n* = 8), *N* = 267, mean age 83.95, 82% men, with incontinence	Individualized nighttime incontinence care (every 2 or 4 h and when awake) and noise abatement and staff feedback to reduce noise for 5 nights	Mixed; Actigraphy; Significant reduction in sleep awakenings with noise and light abatement but not in % sleep or sleep duration. Significant reductions in light events but not noise.	X	X			X	X	

*CHP* Complementary Health Practices, *SPS* Social/Physical Stimulation, *CCP* Clinical Care Practices, *RCT* Randomized controlled trial

### Sites and participants

The 54 studies included 3627 participants residing in nursing homes (*n* = 42), assisted-living facilities (*n* = 11) [21–31], and one elderly residential setting [32]. The facilities were located mostly in the United States (*n* = 25), Europe (*n* = 14) or Asia (*n* = 10). Most studies investigated one (*n* = 23) or two (*n* = 11) long-term care facilities, with a range from 1 to 20. The mean sample size was 66.5 patients (standard deviation [SD] = 58.6), with a range from 5 to 267 participants. The mean age was 81.9 years (SD = 4.4), and the study populations were 41.1% female on average. More than half (52%) of the studies included participants with dementia [23, 24, 27, 33–48], and six studies targeted patients with known sleep problems [28, 30, 49–52].

### Measures

More than half (*n* = 28) of the included studies objectively measured sleep with wrist actigraphy or a daysimeter (measures both light and activity) [53], and one study supplemented these findings with polysomnography [54]. Two studies used polysomnography exclusively [26, 55]. The remainder used self-reporting or reports completed by research or clinical staff, most frequently with the valid and reliable Pittsburgh Sleep Quality Index (*n* = 11) [22, 28, 32, 38, 49, 50, 56–60].

### Quality assessment

The risks of bias for randomized clinical trials and all other quantitative studies are summarized in Tables 5 (*n* = 30) and 6 (*n* = 24), respectively. Risks of bias for individual studies are available in the supplemental materials (Additional file 2: Table S2-BMC and Additional file 3: Table S3-BMC). Among the 30 RCTs, most studies had a low (*n* = 11) or unclear (*n* = 15) risk of selection bias, and a majority (*n* = 28) had a low risk of reporting bias because there was clarity in the reporting of all pre-specified outcomes. Most studies were at low risk for detection (*n* = 21) and attrition (*n* = 19) bias. However, only 7 studies were deemed at low risk for incomplete outcome data for periods longer than 6 weeks because most studies only reported data in the immediate post-intervention period. Regarding performance bias, 9 studies were at high risk because it was not possible to blind anyone to the intervention.

**Table 5:** Summary of Cochrane Risk of Bias for Randomized Controlled Trials [19] (*n* = 30)

	Low Risk	High Risk	Unclear	Not Applicable
Random sequence generation	11	4	15	0
Allocation concealment	11	4	15	0
Blinding of participants and personnel	8	9	13	0
Blinding of outcome assessment	21	2	7	0
Incomplete outcome data (2–6 weeks)	19	5	6	0
Incomplete outcome data (> 6 weeks)	7	0	1	22
Selective reporting	28	0	2	0

For the 24 non-RCTs, most (*n* = 20) studies met at least 12 of the 17 criteria deemed most important for quality appraisal. Many studies, however, did not describe the statistical power of the study (*n* = 19), mention piloting of the intervention (*n* = 13), use valid or reliable measures (*n* = 8), or overstated study conclusions (*n* = 13). Few presented the ethical considerations of the study procedures or intervention (*n* = 19) (Table 6).

**Table 6:** Summary Quantitative Studies and Critical Appraisal Checklist^a^ (*n* = 24)

Criteria	Yes	No
1. Are the aims and objectives of the study clearly stated?	24	0
2. Are the hypotheses and research questions clearly specified?	24	0
3. Are the dependent and independent variables clearly stated?	23	1
4. Have the variables been adequately operationalized?	23	1
5. Is the design of the study adequately described?	24	0
6. Are the research methods appropriate?	22	2
7. Were the instruments used appropriate and adequately tested for reliability and validity?	16	8
8. Is there an adequate description of the source of the sample, inclusion and exclusion criteria, response rates, and (in the case of longitudinal research and post-test in experiments) sample attrition?	20	4
9. Was the statistical power of the study to detect or reject differences (types I and II error) discussed critically?	5	19
10. Are ethical considerations presented?	5	19
11. Was the study piloted?	11	13
12. Were the statistical analyses appropriate and adequate?	22	2
13. Are the results clear and adequately reported?	23	1
14. Does the discussion of the results report them in the light of the hypotheses of the study and other relevant literature?	22	2
15. Are the limitations of the research and its design presented?	20	4
16. Does the discussion generalize and draw conclusion beyond the limits of the data and number and type of people studied?	11	13
17. Can the findings be generalized to other relevant population and time periods?	21	3
18. Are the implications-practical or theoretical-of the research discussed?	12	12
19. Who was the sponsor of the study, and was there a conflict of interest?	11	2 (11 NI)

*NI* Not indicated; ^a^Checklist from Bowling A. Research methods in health: investigating health and health services. 4th ed. Maidenhead Berkshire, England: Open University Press, 2014

### Outcomes

Most studies indicated positive findings (*n* = 24) of non-pharmacological interventions in improving nighttime sleep outcomes [21–28, 30, 32, 34, 39, 42, 44, 48–50, 57–63] whereas 11 studies reported mixed findings (both positive and none) [29, 31, 36, 43, 45–47, 51, 53, 64, 65]. Although reporting other positive outcomes, 19 studies found no change in nighttime sleep quality after the intervention [33, 35, 37, 38, 40, 41, 52, 54–56, 66–74]. The results differed among location sites, with studies conducted in assisted-living facilities reporting a higher proportion of positive findings than those in nursing homes (70 and 35.7%, respectively).

### Study outcomes by intervention type

The interventions employed in the studies varied widely and included interventions in the following categories: clinical care practices (*n* = 3), mind-body practices (*n* = 3), social/physical stimulation (*n* = 11), complementary health practices (*n* = 12), and environmental interventions (*n* = 14). There were a total of 25 individual (same type, though differences in dose) interventions; 15 studies employed either a combination of interventions within a specific category (*n* = 4) or a multicomponent intervention consisting of two or more categories of non-pharmacological intervention (*n* = 11). The following sections summarize the results for each intervention category (Table 3).

### Clinical care practices (*n* = 3) [25, 41, 73]

Practices implemented by nurses included administering a warm evening foot bath to adjust core body temperature [73], providing individualized care (e.g., residents have choice regarding bedtime) [41], and minimizing nighttime disruptions [25]. These interventions had no, mixed, and positive findings, respectively. All three studies used a pre-post design with sample sizes of 30, 33, and 18, respectively, and none of the authors described sample size calculation. All three studies used quasi-experimental designs, with only one including a comparison/control group as well as objectively measuring sleep with actigraphy [73]. Seven multicomponent studies utilized the clinical care practices of minimizing clinical disruptions [44, 45, 54, 65] and/or sleep-wake time management [34, 52, 54, 65]. Among these multicomponent studies, six incorporated clinical care practices to minimize disruptions [44, 45, 54, 65] and/or manage sleep-wake [34, 52, 54, 65].

### Mind-body practices (*n* = 3) [22, 50, 58]

These interventions require some active involvement by the participant, and each of the three studies included only cognitively intact residents. One study was conducted with assisted-living facility residents in Taiwan [22]; the other two were in nursing homes in Egypt [50] and Turkey [58]. Two relaxation strategies had positive results: progressive muscle relaxation [58] and the meditative practice of yoga [22]. This was an adaptation of hatha yoga specifically developed for the reduced flexibility and exercise tolerance of older adults. All three were well-conducted, quasi-experimental studies using the Pittsburgh Sleep Quality Index subjective measure. However, the study examining cognitive-behavioral therapy did not include a comparison group [50]. No mind-body practices were used in the multicomponent studies.

### Social and physical stimulation (*n* = 11) [23, 26, 32, 33, 42, 43, 46, 51, 55, 57, 67]

Eleven studies utilized interventions that prompted participants to engage in a physical or social activity meant to stimulate cognition, mobility, or both. The latter included three RCTs using actigraphy [51] or polysomnography [26, 55] with low risk of bias; however, the findings were not consistent, with positive [26], none [55], and mixed findings [51]. Of three studies testing social and cognitive activities on nursing-home residents with dementia, one reported improved sleep [42], and the other two studies reported mixed findings [43, 46]. The remaining five studies employed physical exercise/activity and varied in quality. Three studies reported improved sleep [23, 32, 57] and two reported no changes in sleep [32, 33]. Six multicomponent studies included physical activity.

### Complementary health practices (*n* = 12) [21, 28–30, 47, 48, 59, 60, 62, 68, 69, 74]

These are interventions that originated outside mainstream medicine, are administered by a practitioner or clinical staff member, and are received by touch, smell, or ingestion. Two studies examining the effect of massage alone [69, 74] did not find improvements in sleep, and one study combining massage with lavender aromatherapy [29] reported mixed findings. Other touch modalities positively improved sleep, including transcutaneous electrical nerve stimulation [48] and therapeutic touch [62]. However, these studies tested only 14 and 6 participants, respectively. Of the four studies of acupressure, three employed a RCT design with low risk of bias [21, 30, 59], while the fourth evaluated 8-h continuous acupressure using a pre-post design with 129 residents [60]. All four reported positive sleep outcomes. The three studies evaluating melatonin use reported no change in sleep (melatonin dose: 8.5 immediate + 1.5 mg sustained release) [68], mixed findings (melatonin dose: 5 mg to 20 mg of melatonin-rich milk) [41], and better sleep (5 mg + magnesium and zinc) [28]. Two multicomponent RCTs included melatonin (2.5 mg and 5 mg) and found positive [27] and no [66] changes in sleep, respectively.

### Environment (*n* = 14) [24, 31, 35–38, 40, 49, 53, 56, 61, 63, 64, 71]

With the exception of one study evaluating the use of a medium-firmness mattress (no improvement in sleep) [56], all studies of the environment focused on increasing light exposure via natural (outdoor) [38, 63] or bright artificial light illumination during the day. The “dose” of light varied considerably from 2500 lx to 10,000 lx administered for 30 min to 8 h per day for 10 days to 10 weeks. It was not possible to correlate dose with findings of no [35–38, 40, 71], positive [24, 49, 61, 63], or mixed [31, 36, 53, 64] improvement in sleep outcomes. Similarly, there was no relationship between study design/risk of bias and outcomes. Light was also included in most (*n* = 8) multicomponent studies, using either natural light [39, 52, 65, 72] or a bright light source of 2500 lx to 10,000 lx [27, 54, 66, 70] for a range of time periods.

### Multicomponent (*n* = 11) [27, 34, 39, 44, 45, 52, 54, 65, 66, 70, 72]

Table 4 provides a summary of the characteristics and components of each multicomponent intervention. These studies included an average of 3 (SD = 1.2) interventions with a range of 2 to 5. All included an environmental intervention: increased light (*n* = 8) [27, 39, 52, 54, 65, 66, 70, 72] and/or reduced noise (*n* = 5) [34, 44, 45, 54, 65]. Three included the use of melatonin [27, 66] or a vitamin B12 supplement [70]. Two studies included 5 interventions (light, noise, activity, fewer disruptions, and sleep-wake management) [54, 65], and two other studies investigated 4 interventions (light, noise, individual care, and sleep-wake management) [44, 45], but the findings were not consistent. Physical activity was investigated in six studies [34, 39, 52, 54, 65, 72], and four studies employed the clinical care practices of individual care [44, 45], fewer disruptions [44, 45, 54, 65], and sleep-wake management [34, 52, 54, 65]. Most studies (*n* = 9) were RCTs, and many of these had a low risk of bias [27, 45, 52, 54, 65]. No clear pattern of intervention combinations emerged among studies with no [52, 54, 66, 70, 72], positive [27, 34, 39, 44], or mixed [45, 65] effect on sleep. The highest proportion of RCTs with several areas of high risk of bias was found among this category of studies [34, 45, 52, 54, 65, 72].

## Discussion

Despite the minimization of the use of physical restraint, the promotion of function-focused care, and the growing trend of culture change in nursing homes, residents spend considerable time inactive, including large amounts of time in bed [75, 76]. Moreover, the institutional environment provides little opportunity for residents to synchronize their circadian clock to the solar day, which is necessary to support alertness during the day and the consolidation of sleep at night. During the night, residents may experience frequent awakenings and fragmented sleep from clinical care practices that increase light and noise. These factors together contribute to the sleep and circadian rhythm disturbances frequently encountered among long-term care residents. In this systematic review of non-pharmacological interventions to improve sleep among long-term care residents, it was found that nearly three-quarters (*n* = 37) of the studies aimed to normalize circadian rhythms by increasing daytime activity (100% of social and physical stimulation category), increasing daytime light (93% of environment category), improving nighttime staff routines to minimize disruptions (67% of clinical care practices), or a combination of these interventions (100% of multicomponent). Although there is sound evidence to support these strategies, the variation in how the interventions were delivered (type of daytime activity or dose of light) reduces the ability to draw definitive conclusions.

Given the functional and cognitive limitations of long-term care residents, it is not surprising that the most frequently studied interventions were largely passive in nature: environmental interventions, complementary health practices, and social/physical stimulation. Daytime light therapy was highly correlated with improved sleep [77], including in those with dementia [78], but more evidence-based guidance is needed regarding dose, delivery, frequency, and duration [79]. Because exposure to natural daylight is often not feasible due to location or building design, supplementing the environment with bright artificial light is a feasible option. Current room lighting systems that eliminate safety concerns (excessive heat or UV rays) can be incorporated in high-use areas such as day and dining rooms. However, this intervention was not found in this review. Other environmental interventions, such as control of ambient temperature with a cooler nighttime temperature, were not found in this review and deserve to be explored in future research [80].

Many single and most multicomponent studies also aimed to “reset” residents’ circadian rhythm with stimulating activities during the day and/or strategies that promote relaxation or deter sleep disruption at night. Fewer than half of those studies that tested either exercise or passive social stimulation reported positive findings [23, 32, 34, 39, 42, 57], although several were well-executed RCTs [26, 39, 57]. Most were conducted by research staff to establish intervention efficacy; thus, translating these time-consuming strategies to current staff levels and roles needs careful consideration. The low number of studies directed toward changing clinical care practices to promote sleep suggests difficulty in altering entrenched routines [52]. These concerns underscore the need to consider intervention feasibility in a low-resource practice environment. A community-participatory approach that actively includes equitable input from long-term care staff, residents, and their families may be needed to overcome challenges to practice change [81]. For example, there is considerable evidence to support acupressure [82], but further research is needed with nursing staff to understand how easily (or not) this practice could be incorporated within their nighttime care routines. Another aspect of feasibility that was absent from the reviewed studies is a cost/benefit analysis, which is needed for buy-in from administrators.

Complementary health practices, although not commonly employed in nursing homes, represented more than a quarter of the included studies and were associated with a high proportion of positive outcomes for both acupressure and melatonin in well-executed studies. Although there were no consistent findings with melatonin in this review, possibly because the dose regimen was quite variable, melatonin is considered a safe and effective approach to improve sleep in older adults [83], including those with dementia [84].

Because some of the studies evaluating mind-body techniques were performed in nursing homes outside the United States, their findings may not be fully generalizable, as their population included more cognitive and physically able residents [50, 58, 75], who could be actively involved in the practice of sleep hygiene principles [50] or self-relaxation techniques [58, 75]. Studies using these interventions may demonstrate better outcomes among the growing population in assisted-living facilities, which has similar characteristics.

The overall assessment of the methodological quality of the included studies revealed that the majority of RCTs were at low risk for most types of risk of bias and most non-RCTs met the standard quality criteria. There were several quality concerns, however, for both study types because we chose to include studies that were underpowered, had a high dropout rate, did not include a control/comparison group, and/or did not collect long-term outcomes.

Also, in some cases, it was difficult to identify which component(s) of a multicomponent intervention contributed to the outcome [85]. These complex interventions, as well as single-component interventions, require considerable resident or staff effort, resulting in participant attrition. Treatment adherence is an important aspect of any proposed intervention, indicating its acceptability to both the participant and the staff. Some interventions require equipment that needs to be maintained by staff and may not be readily available. Correct use of an intervention, whether equipment-based or staff-delivered, necessitates staff/provider training. Also, regular supervision is needed to ensure continued accurate execution, and a quality-improvement program is needed to monitor institution-based outcomes. Intervention integrity was not assessed in this review because only a few studies documented any aspect of treatment fidelity.

## Conclusions

This systematic review located 54 articles evaluating the effects of 25 different non-pharmacological interventions aimed at improving nighttime sleep in long-term care settings. The analysis of these interventions, applied either in isolation or combination, did not reveal a clear pattern of positive findings. Three interventions had the most promising results: increased daytime light exposure (*n* = 21), nighttime use of melatonin (*n* = 6), and acupressure prior to sleep (*n* = 4).

This review highlights the need for further research to help standardize non-pharmacological interventions to improve sleep in institutionalized settings, including dose and timing of light and melatonin use and site of acupressure, and to determine the optimal combination of interventions. Furthermore, more consistent outcome measurements and identification of sub-groups that would best benefit from certain interventions, along with detailed analyses of cost/benefit ratios and feasibility are needed. In summary, non-pharmacological interventions have the potential to improve sleep and circadian rhythm disturbances in residents of long-term care facilities; however, further research is needed to better standardize such interventions and provide clear implementation guidelines using cost-effective practices.

## Additional files

Additional file 1: **Table S1.** Detailed search strategies. Detailed search strategies for each database: Cochrane Library (Wiley), Ovid MEDLINE, Ovid Embase, CINAHL, and Scopus. (PDF 89 kb)

Additional file 2: **Table S2.** Individual study results for Cochrane Risk of Bias for Randomized Controlled Trials [89] (*n* = 30). Provides details (random sequence generation, allocation concealment, blinding of participants and personnel, blinding of outcome assessment, incomplete outcome data, and selective reporting) for individual studies. (DOC 75 kb)

Additional file 3: **Table S3.** Individual studies’ results for the quantitative studies and critical appraisal checklist (*n* = 24). Provides details for each individual study using the nineteen criteria from the Summary Quantitative Studies and Critical Appraisal Checklist. (DOC 109 kb)

## Abbreviations

*RCT*: Randomized controlled trial
*SD*: Standard deviation
