# Supplementary material for: A systematic review of non-pharmacological interventions to improve nighttime sleep among residents of long-term care settings
Source: BMC Geriatr. 2018 Jun 18;18:143. doi: 10.1186/s12877-018-0794-3 (PMC6006939; doi:10.1186/s12877-018-0794-3)
Supplement: Supplementary file 1 — Table S1. Detailed search strategies. Detailed search strategies for each database: Cochrane Library (Wiley), Ovid MEDLINE, Ovid Embase, CINAHL, and Scopus. (PDF 89 kb) [file 12877_2018_794_MOESM1_ESM.pdf]

Supplemental Materials
Table 1 Detailed search strategies

Cochrane Library (Wiley); Searched 12/31/2016

#1 MeSH descriptor: [Sleep] explode all trees
#2 MeSH descriptor: [Sleep Wake Disorders] explode all trees
#3 #1 or #2
#4 sleep* near/2 (quality or quantity or duration or time* or timing or pattern* or rhythm* or promotion or hygiene or efficiency or cycle* or onset or health* or hour* or phase* or support or help or initiat*):ti
#5 sleep* near/2 (disrupt* or disturb* or impair* or interrupt* or depriv* or lack or poor or problem*):ti
#6 (insomnia* or circadian):ti
#7 sleep* near/5 ("biological clock*"):ti
#8 #4 or #5 or #6 or #7
#9 MeSH descriptor: [Hospitalization] this term only
#10 MeSH descriptor: [Hospitals] explode all trees
#11 MeSH descriptor: [Hospital Design and Construction] this term only
#12 MeSH descriptor: [Health Facility Environment] explode all trees
#13 MeSH descriptor: [Hospital Units] explode all trees
#14 MeSH descriptor: [Residential Facilities] explode all trees
#15 MeSH descriptor: [Inpatients] this term only
#16 #9 or #10 or #11 or #12 or #13 or #14 or #15
#17 (hospital* or inpatient* or institutional* or "intensive care" or ward* or hospice* or "nursing home*" or "assisted living" or palliative or "end of life" or "end-of-life" or terminal or "health facilit*" or "residential facilit*" or icu or "critical care"):ti,ab
#18 #3 and #16
#19 MeSH descriptor: [Infant] explode all trees
#20 MeSH descriptor: [Child] explode all trees
#21 #19 or #20
#22 #18 not #21
#23 #8 and #17
#24 (pediatric* or paediatric* or infant* or infancy or newborn* or baby* or babies or neonat* or preterm* or prematur* or child* or schoolchild* or "school age*" or preschool* or kid or kids or toddler or toddlers or adolesc* or pubert* or pubescen* or prepubescen* or teen* or boy* or girl*):ti,ab,kw
#25 #23 not #24
#26 #22 or #25

Epub ahead of print, in-process, and other non-indexed citations, Ovid MEDLINE Daily and Ovid MEDLINE, 1946 to Present; Searched 12/31/2016

1. exp Sleep/
2. exp Sleep Wake Disorders/

3. (sleep$ adj2 (disrupt$ or disturb$ or impair$ or interrupt$ or depriv$ or lack or poor or problem$)).ti.
4. (sleep$ adj2 (quality or quantity or duration or time$ or timing or pattern$ or rhythm$ or promotion or hygiene or efficiency or cycle$ or onset or health$ or hour$ or phase$ or support or help or initiat$)).ti.
5. insomnia$.ti.
6. circadian.ti.
7. (sleep$ adj5 "biological clock$").ti.
8. Hospitalization/
9. exp Hospitals/
10. "Hospital Design and Construction"/
11. exp Health Facility Environment/
12. exp Hospital Units/
13. exp Residential Facilities/
14. Inpatients/
15. (hospital$ or inpatient$ or institutional$ or "intensive care" or ward$ or hospice$ or "nursing home$" or "assisted living" or palliative or "end of life" or "end-of-life" or terminal or "health facilit$" or "residential facilit$" or icu or "critical care").tw.
16. or/1-2
17. or/8-14
18. 16 and 17
19. limit 18 to ("all infant (birth to 23 months)" or "preschool child (2 to 5 years)" or "child (6 to 12 years)")
20. 18 not 19
21. limit 20 to humans
22. or/3-7
23. 22 and 15
24. (pediatric* or paediatric* or infant* or infancy or newborn* or baby* or babies or neonat* or preterm* or prematur* or child* or schoolchild* or "school age*" or preschool* or kid or kids or toddler or toddlers or adolesc* or pubert* or pubescen* or prepubescen* or teen* or boy* or girl*).tw.
25. 23 not 24
26. 20 or 25
27. limit 26 to english language

Ovid Embase, 1974 to 2016 Week 38; Searched 12/31/2016

1. *sleep/ or *sleep quality/ or sleep stage/ or sleep time/
2. sleep deprivation/
3. REM sleep deprivation/
4. hospitalization/
5. *hospital/
6. hospital design/
7. exp ward/
8. residential home/

9. nursing home/
10. assisted living facility/
11. hospice/
12. or/4-11
13. or/1-3
14. 12 and 13
15. limit 14 to (embryo or infant or child or preschool child <1 to 6 years> or school child <7 to 12 years> or adolescent <13 to 17 years>)
16. 14 not 15
17. limit 16 to (human and english language)
18. (sleep$ adj2 (disrupt$ or disturb$ or impair$ or interrupt$ or depriv$ or lack or poor or problem$)).ti.
19. (sleep$ adj2 (quality or quantity or duration or time$ or timing or pattern$ or rhythm$ or promotion or hygiene or efficiency or cycle$ or onset or health$ or hour$ or phase$ or support or help or initiat$)).ti.
20. insomnia$.ti.
21. circadian.ti.
22. (sleep$ adj5 "biological clock$").ti.
23. or/18-22
24. (hospital$ or inpatient$ or institutional$ or "intensive care" or ward$ or hospice$ or "nursing home$" or "assisted living" or palliative or "end of life" or "end-of-life" or terminal or "health facilit$" or "residential facilit$" or icu or "critical care").tw.
25. 23 and 24
26. (pediatric* or paediatric* or infant* or infancy or newborn* or baby* or babies or neonat* or preterm* or prematur* or child* or schoolchild* or "school age*" or preschool* or kid or kids or toddler or toddlers or adolesc* or pubert* or pubescen* or prepubescen* or teen* or boy* or girl*).tw.
27. 25 not 26
28. limit 27 to english language
29. 17 or 28

CINAHL (EBSCOhost), 1981 to present; Searched 12/31/2016

S22 S19 NOT S20 Narrow by Language: - english
S21 S19 NOT S20
S20 TX pediatric* or paediatric* or infant* or infancy or newborn* or baby* or babies or neonat* or preterm* or prematur* or child* or schoolchild* or "school age*" or preschool* or kid or kids or toddler or toddlers or adolesc* or pubert* or pubescen* or prepubescen* or teen* or boy* or girl*
S19 S17 AND S18
S18 S8 OR S9 OR S10 OR S11 OR S12 OR S13 OR S14 OR S15 OR S16
S17 S1 OR S2 OR S3 OR S4 OR S5 OR S6 OR S7
S16 AB hospital* OR inpatient* or institutional* OR "intensive care" OR ward* OR hospice* OR "nursing home*" OR "assisted living" OR palliative OR "end of life" OR "end-of-life" OR terminal OR "health facilit*" OR "residential facilit*" OR icu OR "critical care"

S15 TI hospital* OR inpatient* or institutional* OR "intensive care" OR ward* OR hospice* OR "nursing home*" OR "assisted living" OR palliative OR "end of life" OR "end-of-life" OR terminal OR "health facilit*" OR "residential facilit*" OR icu OR "critical care"
S14 (MH "Inpatients") OR (MH "Nursing Home Patients") OR (MH "Aged, Hospitalized") OR (MH "Critically Ill Patients") OR (MH "Terminally Ill Patients+")
S13 (MH "Residential Facilities+")
S12 (MH "Hospital Units+")
S11 (MH "Health Facility Environment")
S10 (MH "Nursing Home Design and Construction") OR (MH "Hospital Design and Construction")
S9 (MH "Hospitals+")
S8 (MH "Hospitalization")
S7 TI sleep* N5 "biological clock*"
S6 TI circadian
S5 TI insomnia
S4 TI sleep* N2 (quality OR quantity OR duration OR time* OR timing OR pattern* OR rhythm* OR promotion OR hygiene OR efficiency OR cycle* OR onset OR health* OR hour* OR phase* OR support OR help OR initiat*)
S3 TI sleep* N2 (disrupt* OR disturb* OR impair* OR interrupt* OR depriv* OR lack OR poor OR problem*)
S2 (MH "Sleep Disorders+")
S1 (MH "Sleep+")

Scopus (Elsevier), 2003 to present; 12/31/2016

((((TITLE (sleep* W/2 (disrupt* OR disturb* OR impair* OR interrupt* OR depriv* OR lack OR poor OR problem*))) OR (TITLE (sleep* W/2 (quality OR quantity OR duration OR time* OR timing OR pattern* OR rhythm* OR promotion OR hygiene OR efficiency OR cycle* OR onset OR health* OR hour* OR phase* OR support OR help OR initiat*)))) AND (TITLE-ABS-KEY (hospital* OR inpatient* OR institutional* OR "intensive care" OR ward* OR hospice* OR "nursing home*" OR "assisted living" OR palliative OR "end of life" OR "end-of-life" OR terminal OR "health facilit*" OR "residential facilit*" OR icu OR "critical care"))) AND NOT (TITLE-ABS-KEY (pediatric* OR paediatric* OR infant* OR infancy OR newborn* OR baby* OR babies OR neonat* OR preterm* OR prematur* OR child* OR schoolchild* OR "school age*" OR preschool* OR kid OR kids OR toddler OR toddlers OR adolesc* OR pubert* OR pubescen* OR prepube))) AND (LANGUAGE (english))
