# Supplementary material for: A systematic review of non-pharmacological interventions to improve nighttime sleep among residents of long-term care settings
Source: BMC Geriatr. 2018 Jun 18;18:143. doi: 10.1186/s12877-018-0794-3 (PMC6006939; doi:10.1186/s12877-018-0794-3)
Supplement: Supplementary file 2 — Table S2. Individual study results for Cochrane Risk of Bias for Randomized Controlled Trials [89] (n = 30). Provides details (random sequence generation, allocation concealment, blinding of participants and personnel, blinding of outcome assessment, incomplete outcome data, and selective reporting) for individual studies. (DOC 75 kb) [file 12877_2018_794_MOESM2_ESM.doc]

***Supplemental Materials***

**Table 2. Individual Studies - Cochrane Risk of Bias for RCTs* (n=30)**

| * | Reference | Random Sequence Generation  N= 4 High Risk | Allocation Concealment  N=4 High Risk | Blinding of Participants and Personnel  N=9 High Risk | Blinding of Outcome Assessment  N=2 High Risk | Incomplete Outcome Data (2-6 weeks)  N=5 High Risk | Incomplete Outcome Data (> 6 weeks)  N=0 High Risk | Selective Reporting  N=0 High Risk |
| --- | --- | --- | --- | --- | --- | --- | --- | --- |
| SPS | Chen (2015)^57^ | Low Risk | Low Risk | Unclear | Low Risk | Unclear | Low Risk | Low Risk |
| SPS | Eggermont (2010)^67^ | Unclear | Unclear | Unclear | Low Risk | Low Risk | NA | Low Risk |
| SPS | Kuck (2014)^51^ | Low Risk | Low Risk | **High Risk** | Unclear | Low Risk | NA | Low Risk |
| SPS | Lorenz (2012)^55^ | Low Risk | Low Risk | **High Risk** | Low Risk | Low Risk | Low Risk | Low Risk |
| SPS | Richards (2005)^43^ | Unclear | Unclear | **High Risk** | Unclear | Low Risk | NA | Low Risk |
| SPS | Richards (2011)^26^ | Low Risk | Low Risk | Unclear | Low Risk | Unclear | Low Risk | Low Risk |
| CHP | Chen (1999)^21^ | Unclear | Unclear | Unclear | Low Risk | Low Risk | NA | Low Risk |
| CHP | Gehrman (2009)^68^ | Unclear | Low Risk | Low Risk | Low Risk | Low Risk | NA | Low Risk |
| CHP | Harris (2012)^69^ | Low Risk | Low Risk | Unclear | Unclear | Unclear | NA | Low Risk |
| CHP | Nelson (2010)^74^ | Unclear | Unclear | **High Risk** | Unclear | Low Risk | NA | Low Risk |
| CHP | Reza (2010)^59^ | Unclear | Low Risk | Low Risk | Low Risk | Low Risk | NA | Low Risk |
| CHP | Rondanelli (2011)^28^ | Unclear | Unclear | Low Risk | Low Risk | Low Risk | NA | Low Risk |
| CHP | Soden (2004)^29^ | Low Risk | Low Risk | Low Risk | Low Risk | Low Risk | NA | Unclear |
| CHP | Sun (2010)^30^ | Low Risk | Low Risk | Low Risk | Low Risk | Low Risk | Low Risk | Unclear |
| CHP | Van Someren (1998)^48^ | Unclear | Unclear | Low Risk | Unclear | **High Risk** | NA | Low Risk |
| ENV | Ancoli-Israel (2002)^36^ | **High Risk** | **High Risk** | Unclear | Low Risk | **High Risk** | NA | Low Risk |
| ENV | Ancoli-Israel (2003)^35^ | **High Risk** | **High Risk** | Unclear | Low Risk | Low Risk | NA | Low Risk |
| ENV | Burns (2009)^37^ | Low Risk | Low Risk | Unclear | Low Risk | Low Risk | NA | Low Risk |
| ENV | Calkins (2007)^38^ | Unclear | Unclear | Unclear | Low Risk | Low Risk | NA | Low Risk |
| ENV | Dowling (2005)^40^ | Unclear | Unclear | Unclear | Low Risk | Unclear | NA | Low Risk |
| ENV | Lyketsos (1999)^24^ | Unclear | Unclear | Unclear | Low Risk | Low Risk | NA | Low Risk |
| MC | Alessi (1999)^34^ | Unclear | Unclear | **High Risk** | Unclear | Unclear | Low Risk | Low Risk |
| MC | Alessi (2005)65 | Low Risk | **High Risk** | **High Risk** | Low Risk | Low Risk | NA | Low Risk |
| MC | Connell (2007)^39^ | Unclear | Unclear | Unclear | Low Risk | Low Risk | NA | Low Risk |
| MC | Dowling (2008)^66^ | Unclear | Unclear | Low Risk | Low Risk | Unclear | Low Risk | Low Risk |
| MC | Gammack (2009)^72^ | **High Risk** | **High Risk** | Unclear | **High Risk** | **High Risk** | NA | Low Risk |
| MC | Martin (2007)^52^ | Low Risk | Unclear | **High Risk** | **High Risk** | **High Risk** | NA | Low Risk |
| MC | Ouslander (2006)^54^ | **High Risk** | Unclear | **High Risk** | Low Risk | **High Risk** | NA | Low Risk |
| MC | Riemersma-Van Der Lek (2008)^27^ | Low Risk | Low Risk | Low Risk | Low Risk | Low Risk | Low Risk | Low Risk |
| MC | Schnelle (1999)^45^ | Unclear | Unclear | **High Risk** | Unclear | Low Risk | NA | Low Risk |

Higgins JP, Altman DG, Gøtzsche PC, Jüni P, Moher D, Oxman AD, Savović J, Schulz KF, Weeks L, Sterne JA. The Cochrane Collaboration’s tool for assessing risk of bias in randomised trials. BMJ. 2011 Oct 18;343:d5928. https://doi.org/10.1136/bmj.d5928

* Category of Interventions:

CCP = Clinical Care Practices

MBP = Mind-Body Practices

SPS = Social and Physical Stimulation

CHP = Complementary Health Practices

ENV = Environment

MC = Multi-Component
