# Supplementary material for: A systematic review of non-pharmacological interventions to improve nighttime sleep among residents of long-term care settings
Source: BMC Geriatr. 2018 Jun 18;18:143. doi: 10.1186/s12877-018-0794-3 (PMC6006939; doi:10.1186/s12877-018-0794-3)
Supplement: Supplementary file 3 — Table S3. Individual studies’ results for the quantitative studies and critical appraisal checklist (n = 24). Provides details for each individual study using the nineteen criteria from the Summary Quantitative Studies and Critical Appraisal Checklist. (DOC 109 kb) [file 12877_2018_794_MOESM3_ESM.doc]

***Supplemental Materials***

**Table 3. Individual Studies – Quantitative studies and critical appraisal checklist * (n=24)**

|  |  | **1**  **N=0** | **2**  **N=0** | **3**  **N=0** | **4**  **N=1** | **5**  **N=0** | **6**  **N=2** | **7**  **N=8** | **8**  **N=4** | **9**  **N= 19** | **10**  **N= 19** | **11**  **N= 13** | **12**  **N=2** | **13**  **N=1** | **14**  **N=2** | **15**  **N=4** | **16**  **N=13** | **17**  **N=3** | **18**  **N= 12** | **19**  **N=2** |
| --- | --- | --- | --- | --- | --- | --- | --- | --- | --- | --- | --- | --- | --- | --- | --- | --- | --- | --- | --- | --- |
| CCP | Kim (2016)^73^ | Yes | Yes | Yes | Yes | Yes | Yes | Yes | Yes | **No** | **No** | Yes | Yes | Yes | Yes | Yes | Yes | Yes | **No** | Yes |
| CCP | Matthews (1996)^41^ | Yes | Yes | Yes | Yes | Yes | Yes | Yes | Yes | **No** | **No** | Yes | Yes | Yes | Yes | Yes | Yes | Yes | **No** | Yes |
| CCP | O’Rourke (2001)^25^ | Yes | Yes | Yes | Yes | Yes | Yes | Yes | Yes | **No** | Yes | **No** | Yes | Yes | Yes | Yes | **No** | Yes | Yes | NI |
| MBP | Chen (2010)^22^ | Yes | Yes | Yes | Yes | Yes | Yes | Yes | Yes | **No** | **No** | **No** | Yes | Yes | Yes | Yes | **No** | Yes | Yes | NI |
| MBP | El Kady (2012)^50^ | Yes | Yes | Yes | Yes | Yes | Yes | Yes | Yes | **No** | **No** | **No** | Yes | Yes | Yes | Yes | **No** | Yes | Yes | NI |
| MBP | Örsal (201e)^58^ | Yes | Yes | Yes | Yes | Yes | Yes | Yes | Yes | **No** | **No** | Yes | Yes | Yes | **No** | Yes | Yes | Yes | **No** | Yes |
| SPS | Alessi (1995)^33^ | Yes | Yes | Yes | Yes | Yes | Yes | **No** | Yes | Yes | **No** | **No** | Yes | Yes | Yes | **No** | **No** | Yes | Yes | NI |
| SPS | Lee (2008)^23^ | Yes | Yes | Yes | Yes | Yes | Yes | **No** | Yes | **No** | Yes | Yes | Yes | Yes | Yes | Yes | **No** | **No** | Yes | NI |
| SPS | Richards (2001)^42^ | Yes | Yes | Yes | Yes | Yes | **No** | Yes | **No** | **No** | **No** | **No** | Yes | Yes | Yes | Yes | Yes | Yes | **No** | Yes |
| SPS | Taboonpong (2010)^32^ | Yes | Yes | Yes | Yes | Yes | Yes | Yes | Yes | **No** | **No** | Yes | Yes | Yes | Yes | Yes | Yes | Yes | **No** | Yes |
| SPS | Thodberg (2015)^46^ | Yes | Yes | Yes | Yes | Yes | Yes | Yes | Yes | Yes | Yes | Yes | Yes | Yes | Yes | Yes | Yes | Yes | **No** | Yes |
| CHP | Braun (1986)^62^ | Yes | Yes | Yes | **No** | Yes | Yes | **No** | **No** | **No** | **No** | **No** | **No** | **No** | Yes | **No** | **No** | Yes | Yes | NI |
| CHP | Simoncini (2015)^60^ | Yes | Yes | Yes | Yes | Yes | Yes | Yes | Yes | Yes | **No** | Yes | Yes | Yes | Yes | Yes | Yes | Yes | N**o** | Yes |
| CHP | Valtonen (2005)^47^ | Yes | Yes | Yes | Yes | Yes | Yes | Yes | Yes | **No** | **No** | Yes | Yes | Yes | Yes | Yes | Yes | Yes | **No** | Yes |
| ENV | Akyar (2013)^49^ | Yes | Yes | Yes | Yes | Yes | Yes | **No** | Yes | Yes | Yes | **No** | Yes | Yes | Yes | Yes | **No** | Yes | Yes | NI |
| ENV | Ancuelle (2015)^56^ | Yes | Yes | Yes | Yes | Yes | Yes | Yes | Yes | Yes | Yes | No | Yes | Yes | Yes | Yes | **No** | Yes | Yes | **No** |
| ENV | Castor (1991)^63^ | Yes | Yes | Yes | Yes | Yes | Yes | **No** | Yes | **No** | **No** | **No** | **No** | Yes | Yes | Yes | **No** | **No** | Yes | NI |
| ENV | Fetveit (2003)^61^ | Yes | Yes | Yes | Yes | Yes | Yes | Yes | Yes | **No** | **No** | **No** | Yes | Yes | Yes | Yes | **No** | Yes | Yes | NI |
| ENV | Fetveit (2004)^64^ | Yes | Yes | Yes | Yes | Yes | Yes | Yes | Yes | **No** | **No** | **No** | Yes | Yes | Yes | Yes | **No** | Yes | Yes | NI |
| ENV | Figueiro (2014)^53^ | Yes | Yes | Yes | Yes | Yes | Yes | **No** | Yes | **No** | **No** | **No** | Yes | Yes | Yes | **No** | **No** | Yes | Yes | **No** |
| ENV | Koyama (1999)^71^ | Yes | Yes | **No** | Yes | Yes | **No** | **No** | **No** | **No** | **No** | **No** | Yes | Yes | **No** | Yes | Yes | Yes | **No** | Yes |
| ENV | Wu (2015)^31^ | Yes | Yes | Yes | Yes | Yes | Yes | Yes | Yes | **No** | **No** | Yes | Yes | Yes | Yes | Yes | Yes | Yes | **No** | Yes |
| MC | Ito (2001)^70^ | Yes | Yes | Yes | Yes | Yes | Yes | **No** | **No** | **No** | **No** | **No** | Yes | Yes | Yes | **No** | **No** | **No** | **No** | NI |
| MC | Schnelle (1998)^44^ | Yes | Yes | Yes | Yes | Yes | Yes | Yes | Yes | **No** | **No** | Yes | Yes | Yes | Yes | Yes | Yes | Yes | **No** | Yes |

CCP = Clinical Care Practices

MBP = Mind-Body Practices

SPS = Social and Physical Stimulation

CHP = Complementary Health Practices

ENV = Environment

MC = Multi-Component

| **#** | **Criteria** |
| --- | --- |
| 1 | Are the aims and objectives of the study clearly stated? |
| 2 | Are the hypotheses and research questions clearly specified? |
| 3 | Are the dependent and independent variables clearly stated? |
| 4 | Have the variables been adequately operationalized? |
| 5 | Is the design of the study adequately described? |
| 6 | Are the research methods appropriate? |
| 7 | Were the instruments used appropriate and adequately tested for reliability and validity? |
| 8 | Is there an adequate description of the source of the sample, inclusion and exclusion criteria, response rates, and (in the case of longitudinal research and post-test in experiments) sample attrition? |
| 9 | Was the statistical power of the study to detect or reject differences (types I and II error) discussed critically? |
| 10 | Are ethical considerations presented? |
| 11 | Was the study piloted? |
| 12 | Were the statistical analyses appropriate and adequate? |
| 13 | Are the results clear and adequately reported? |
| 14 | Does the discussion of the results report them in the light of the hypotheses of the study and other relevant literature? |
| 15 | Are the limitations of the research and its design presented? |
| 16 | Does the discussion generalize and draw conclusion beyond the limits of the data and number and type of people studied? |
| 17 | Can the findings be generalized to other relevant population and time periods? |
| 18 | Are the implications-practical or theoretical-of the research discussed? |
| 19 | Who was the sponsor of the study, and was there a conflict of interest? |

Bowling, A. (2014). *Research methods in health: investigating health and health services* (4th ed.). Open University Press.
